# 中国非小细胞肺癌免疫检查点抑制剂治疗专家共识（2019年版）

**DOI:** 10.3779/j.issn.1009-3419.2020.02.01

**Published:** 2020-02-20

**Authors:** 彩存 周, 洁 王, 宏 步, 宝成 王, 宝惠 韩, 铀 卢, 哲海 王, 波 朱, 子平 王, 启斌 宋, 胜祥 任, 冬梅 林, 雅億 何, 晓桦 胡, 洪云 赵, 叔逵 秦

**Affiliations:** 1 200433 上海，同济大学附属上海市肺科医院 Shanghai Pulmonary Hospital Affiliated to Tongji University, Shanghai 200433, China; 2 100021 北京，中国医学科学院肿瘤医院 Cancer Hospital Chinese Academy of Medical Sciences, Beijing 100021, China; 3 610041 成都，四川大学华西医院 West China Hospital, Sichuan University, Chengdu 610041, China; 4 250031 济南，解放军960医院 NO. 960 Hospital of PLA, Jinan 250031, China; 5 200030 上海，上海市胸科医院 Shanghai Chest Hospital, Shanghai 200030, China; 6 250117 济南，山东省肿瘤医院 Shandong Caner Hospital and Institute, Jinan 250117, China; 7 400042 重庆，重庆大坪医院 Chongqing Daping Hospital, Chongqing 400042, China; 8 100142 北京，北京大学肿瘤医院 Beijing Cancer Hospital, Beijing 100142, China; 9 430060 武汉，武汉大学人民医院 Renmin Hospital of Wuhan University, Wuhan 430060, China; 10 530021 南宁，广西医科大学第一附属医院 The First Affiliated Hospital of Guangxi Medical University, Nanning 530021, China; 11 510060 广州，中山大学肿瘤防治中心 Sun Yat-sen University Cancer Center, Guangzhou 510060, China; 12 210002 南京，中国人民解放军东部战区总医院秦淮医疗区 Qinhuai Medical Area, Eastern Theater General Hospital of PLA China, Nanjing 210002, China

**Keywords:** 肺肿瘤, 肿瘤免疫治疗, 免疫检查点抑制剂, PD-1/PD-L1, Lung neoplasms, Cancer immunotherapy, Immune checkpoint inhibitor, PD-1/PD-L1

## Abstract

非小细胞肺癌（non-small cell lung cancer, NSCLC）是肺癌中最常见的病理类型，大多数NSCLC患者在确诊时已属晚期。对于驱动基因突变阴性的患者而言，目前的治疗仍以化疗为主，总体预后较差，改善治疗现状、获得长期生存是晚期NSCLC患者最迫切的需求。近年来，肿瘤免疫治疗发展迅速，免疫检查点抑制剂（immune checkpoint inhibitors, ICIs），尤其是以程序性死亡因子-1（programmed death-1, PD-1）/程序性死亡因子配体-1（programmed death-ligand 1, PD-L1）为靶点的ICIs在驱动基因突变阴性的NSCLC治疗中取得了突破性的进展，为患者带来了生存获益，改变了NSCLC的治疗格局，显示出越来越重要的地位。由中国临床肿瘤学会（Chinese society of clinical oncology, CSCO）NSCLC专家委员会牵头，组织该领域的相关专家，在参考国内外文献、系统评价中外临床研究结果、结合专家经验与体会的基础上，达成统一意见并制定本共识，以期指导国内同行更好地应用ICIs治疗NSCLC。

## Abstract (en)

Non-small cell lung cancer (NSCLC) is the most common pathological type of lung cancer, most NSCLC patients are at advanced stage at the time of diagnosis. For patients without sensitive driven-oncogene mutations, chemotherapy is still the main treatment at present, the overall prognosis is poor. Improving outcomes and obtaining long-term survival are the most urgent needs of patients with advanced NSCLC. In recent years, immunotherapy has developed rapidly. Immune checkpoint inhibitors (ICIs), especially targeting programmed death-1 (PD-1)/programmed death-ligand 1 (PD-L1), have made a breakthrough in the treatment of NSCLC, beneficial to patients' survival and changed the treatment pattern for NSCLC. It shows more and more important role in the treatment of NSCLC. Led by NSCLC expert committee of Chinese society of clinical oncology (CSCO), relevant experts in this field were organized. On the basis of referring to domestic and foreign literature, systematically evaluating the results of Chinese and foreign clinical trials, and combining the experiences of the experts, the experts group reached an agreement to develop this consensus. It will guide domestic counterparts for better application of ICIs to treat NSCLC.

## 1 前言

肺癌是全球发病率和致死率最高的恶性肿瘤，据2018年全球肿瘤统计分析报告显示，全球肺癌的男女发病率分别为：年龄标化率（age standardized rate, ASR）1.5/10万和14.6/10万; 死亡率为ASR 27.1/10万和11.2/10万^[1]^。按照病理组织学分类，肺癌可分为小细胞肺癌和非小细胞肺癌（non-small cell lung cancer, NSCLC）两大类。其中NSCLC作为最常见的肺癌组织学类型，占到所有肺癌的85%，其5年生存率仅为16%^[2]^。含铂双药作为驱动基因突变阴性的晚期NSCLC患者的传统治疗方案，其中位无进展生存期（median progression free survival, mPFS）与中位总生存期（median overall survival, mOS）分别为5个月-6个月和11个月-12个月，患者的远期疗效亟待提高^[3]^。免疫治疗，特别是免疫检查点抑制剂（immune checkpoint inhibitors, ICIs）作为一种全新的抗肿瘤疗法自20世纪90年代末问世以来，已在包括肺癌在内的多个肿瘤治疗领域取得了突破性的进展，2013年被《Science》杂志评为年度十大科学突破之首^[4]^，而发现免疫疗法的James P Alison和Tasuku Honjo也因为在肿瘤免疫领域的突出贡献，荣获2018年诺贝尔生理学或医学奖。随着美国食品药品监管局（Food and Drug Administration, FDA）与中国国家药品监督管理局（National Medical Products Administration, NMPA）相继批准ICIs用于肺癌治疗，免疫治疗为晚期NSCLC的治疗带来了新希望。然而NSCLC的免疫治疗特别是ICIs的临床应用，在国内刚刚起步，在如何选择优势人群、确定治疗方案、疗效评估、不良反应的处理以及药物使用禁忌症等方面尚缺乏经验。为了更好地指导国内临床工作者正确使用免疫治疗，特别是ICIs，中国临床肿瘤学会（Chinese Society of Clinical Oncology, CSCO）NSCLC专家委员会牵头，组织该领域的相关专家，在参考国内外文献、系统评价中外临床研究结果、结合专家经验与体会的基础上，达成统一意见并制定本《中国非小细胞肺癌免疫治疗专家共识（2019年版）》，供国内同行参考，以期进一步规范和指导NSCLC免疫治疗的临床实践。

## 2 肿瘤的免疫逃逸机制

### 2.1 机体正常的免疫监视

在正常生理状态下，机体免疫系统具有识别“自己”抗原和“异己”抗原的能力，在识别了“异己”抗原后，免疫系统将会被激活并杀伤“异己”。在肿瘤产生之初，肿瘤细胞会释放特异性的肿瘤相关抗原，树突状细胞等抗原递呈细胞（antigen presenting cells, APCs）识别摄取并加工肿瘤抗原，ACPs进入淋巴组织激活T细胞，T细胞被激活后迁移至肿瘤部位，渗透至肿瘤组织，T细胞通过特异性的受体识别并杀死肿瘤细胞，而肿瘤细胞凋亡后又会表达和释放更多的肿瘤相关抗原，进一步激活更多的T细胞以维持机体有效的免疫监视，避免机体内肿瘤的发生^[5]^。

### 2.2 肿瘤的免疫逃逸机制和相应的治疗策略

正常情况下，肿瘤的发生发展过程中必然会累积众多突变，这些不同的突变会编码众多“异己”抗原，使得产生突变的肿瘤细胞被免疫系统识别并清除。但是，肿瘤细胞在与免疫系统的抗争过程中，可以获得多种逃脱免疫系统监视的方法，最终导致肿瘤的发生。肿瘤的免疫逃逸机制，大致可分为以下三个方面^[6]^：①免疫原性丧失：肿瘤细胞虽能表达某些特殊抗原，可以被免疫系统识别，但是这些肿瘤细胞可以通过其他一些分子（如免疫检查点）的表达，起到抑制免疫系统的作用。在这种情况下，虽然免疫系统可以识别肿瘤细胞，却不能被有效激活，不能发挥杀死肿瘤细胞的作用。针对肿瘤细胞免疫原性的丧失，以PD-1/PD-L1单抗为代表的ICIs疗法已经改变了肿瘤治疗的格局。ICIs能够抑制T细胞表面的PD-1与肿瘤细胞表面的PD-L1配体结合，再次激活T细胞，发挥杀死肿瘤细胞的作用。②抗原性丧失：肿瘤细胞通过丧失特殊抗原的表达来避开免疫系统的识别，从而躲避免疫系统的杀伤。针对肿瘤细胞表面抗原的丧失，具有代表性的免疫疗法是嵌合抗原受体T细胞（chimeric antigen receptor T-cell, CAR-T）治疗，即通过细胞工程技术改造免疫细胞，使之可以识别肿瘤细胞表面的其他特定“异己”抗原而杀死肿瘤细胞。CAR-T治疗已在血液肿瘤领域取得了重大进展^[7]^，但在实体瘤领域，CAR-T治疗尚未取得实质性进展。③免疫抑制微环境：虽然在实体肿瘤组织中浸润的免疫细胞在体外环境下仍能发挥有效作用，但在实体肿瘤组织内，存在多种负性调节的细胞和细胞因子，共同构成了肿瘤组织周围的免疫抑制的微环境，阻止免疫系统发挥正常的杀死肿瘤细胞的作用。针对免疫抑制微环境，可通过干扰肿瘤微环境中存在的多种抑制免疫反应的细胞因子来实现肿瘤免疫治疗的目的。如利用间变性淋巴瘤激酶5（anaplastic lymphoma kinase, ALK-5）抑制剂抑制肿瘤转移^[8]^，目前尚处于临床研究阶段。

**Figure:**
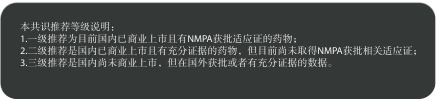


## 3 免疫检查点抑制剂

T细胞活化时，相应共抑制信号通路的免疫检查点（CTLA-4, PD-1/PD-L1）的表达会增加，而ICIs通过阻断上述检查点恢复或增强机体的抗肿瘤免疫^[9]^。

### 3.1 CTLA-4（cytotoxic T lymphocyte antigen 4, CTLA-4）抗体

CTLA-4是由*CTLA-4*基因编码的一种跨膜蛋白质，表达在活化的CD4^+^和CD8^+^T细胞，与配体CD80（B7-1）和CD86（B7-2）结合。CTLA-4能够中止已激活的T细胞的反应（T cell response, TCR）以及介导调节性T细胞（mediates regulatory T cells, Treg）的抑制功能; 还能介导树突细胞结合CD80/CD86并诱导色氨酸降解酶IDO的表达，从而导致TCR的抑制。CTLA-4抗体正是通过与CTLA-4的结合来减少Treg的抑制，激活TCR^[10]^。目前国内尚无CTLA-4抗体获批上市，国外已经获批上市的CTLA-4抗体有百时美施贵宝公司的伊匹木单抗（Ipilimumab），2011年获得FDA批准用于治疗晚期黑色素瘤。

### 3.2 程序性死亡因子1（programmed death 1, PD-1）/程序性死亡因子配体-1（programmed death-ligand 1, PD-L1）抗体

PD-1是表达在T细胞表面的一种重要的免疫抑制跨膜蛋白，其配体为PD-L1。在肿瘤的微环境中，肿瘤细胞能够表达PD-L1，与PD-1结合，减少TCR信号通路的磷酸化，降低TCR通路下游的信号激活以及T细胞的激活和细胞因子的生成，因此抑制PD-1通路可以加速和增强机体的自身免疫^[10]^。PD-1/PD-L1抗体正是通过与PD-1/PD-L1的结合来阻断该通路，恢复机体对肿瘤细胞的免疫杀伤功能。目前国内获批上市的PD-1抗体有：百时美施贵宝公司的纳武利尤单抗（Nivolumab，商品名Opdivo“欧狄沃”）; 默沙东公司的帕博利珠单抗（Pembrolizumab，商品名“可瑞达”）; 君实的JS-001（商品名“拓益”）; 信达公司的信迪利单抗（Sintilimab，商品名“达伯舒”）; 恒瑞公司的卡瑞丽珠单抗（Camrelizumab，商品名“艾瑞卡”）以及百济神州公司的替雷利珠单抗（Tiselizumab，商品名“百泽安”）。国内获批上市的PD-L1抗体为阿斯利康公司的度伐利尤单抗（Durvalumab，商品名“英飞凡”）。国外上市的PD-L1抗体有罗氏公司的阿特珠单抗（Atezolizumab）、辉瑞和默克公司联合推出的Bavencio以及阿斯利康公司的度伐利尤单抗。

## 4 非小细胞肺癌的免疫治疗

### 4.1 驱动基因突变阴性非小细胞肺癌

#### 4.1.1 晚期NSCLC一线免疫治疗

见表 1。

**1:** 晚期NSCLC一线免疫治疗 First-line immunotherapy for advanced NSCLC

分层	一级推荐	二级推荐	三级推荐
PD-L1≥50%	帕博利珠单抗单药		
1%≤PD-L1≤49%	鳞癌：帕博利珠单抗单药	鳞癌：帕博利珠单抗联合铂类+紫杉类	
	非鳞癌：帕博利珠单抗单药或帕博利珠单抗联合铂类+培美曲塞	非鳞癌：帕博利珠单抗单药	
PD-L1 < 1%或者未知	非鳞癌：帕博利珠单抗联合铂类+培美曲塞	鳞癌：帕博利珠单抗联合铂类+紫杉类	非鳞癌：阿特珠单抗联合贝伐珠单抗联合化疗（卡铂和紫杉醇）
NSCLC: non-smal l cel l lung cancer; PD-1: programmed death 1.			

主要证据来源：（1）单药PD-L1 ≥50%：帕博利珠单抗单药（KN-024研究），FDA批准; （2）单药PD-L1≥1%：帕博利珠单抗单药（KN-042研究）; （3）免疫联合化疗：不论PD-L1表达：非鳞癌：帕博利珠单抗联合培美/铂类（KN-021G，KN-189研究），FDA与NMPA批准; 鳞癌：帕博利珠单抗联合卡铂与紫杉醇/白蛋白紫杉醇（KN-407研究）; （4）免疫联合化疗和抗血管：阿特珠单抗+贝伐珠单抗+紫杉醇+卡铂（IMpower 150研究）。

KEYNOTE-024研究结果显示帕博利珠单抗在PD-L1表达≥50%的驱动基因阴性晚期NSCLC人群中，与标准的含铂化疗相比，使用帕博利珠单抗的患者其PFS（HR为0.50）与OS（HR为0.60）都得到了显著的改善，且任何级别的治疗相关不良事件（73% *vs* 90%）和≥3级的不良事件发生率少于化疗组（27% *vs* 53%）^[11]^。基于KEYNOTE-024研究，2016年FDA批准帕博利珠单抗用于PD-L1≥50%的驱动基因阴性晚期NSCLC的一线治疗。在此研究基础之上，KEYNOTE-042研究进一步探索了在PD-L1表达≥50%、≥20%和≥1%的NSCLC患者帕博利珠单抗单药一线治疗的效果。结果显示帕博利珠单抗组的mOS均优于单独化疗组，其中PD-L1≥50%的人群的疗效最为显著^[12]^。KEYNOTE-042临床研究将帕博利珠单抗治疗的优势人群由PD-L1≥50%扩展至PD-L1≥1%的驱动基因阴性晚期NSCLC人群，扩大了PD-1治疗的获益人群，中国人群数据与全球数据保持一致。基于KEYNOTE-042的研究结果，2019年FDA和NMPA批准了帕博利珠单抗作为单一疗法，用于一线治疗PD-L1≥1%、人表皮生长因子受体（epidermal growth factor receptor, EGFR）/ALK阴性晚期NSCLC患者。

KEYNOTE-021G研究对晚期非鳞癌NSCLC患者评估了帕博利珠单抗联合培美曲塞/卡铂对比单纯培美曲塞/卡铂的疗效，研究获得了阳性结果^[13]^。基于KeyNote-021G的研究，Ⅲ期的KEYNOTE-189临床试验探索晚期无突变基因的非鳞癌NSCLC患者，采用帕博利珠单抗联合培美曲塞/铂类相比于传统的培美曲塞/铂类联合方案的疗效。经过10.5个月的随访，联合治疗组在各方面都展现出了压倒性的优势：一年生存率69.2% *vs* 49.4%，降低死亡风险50%;mPFS为8.8个月*vs* 4.9个月，降低48%的疾病进展风险; 治疗有效率47.6% *vs* 18.9%，提高了2.5倍; 两种治疗方案的副作用相当，≥3级的副作用比例分别是67.2% *vs* 65.8%，基本都是可控。值得一提的是：不管PD-L1表达水平的高低，联合组的患者生存期均明显延长^[14]^。基于上述结果FDA及NMPA均批准了帕博利珠单抗联合含铂双药一线治疗晚期无突变基因的非鳞癌NSCLC患者。

KEYNOTE-407研究采用帕博利珠单抗联合卡铂与紫杉醇或白蛋白紫杉醇对比化疗一线治疗晚期鳞癌NSCLC患者的疗效。与单纯化疗相比，帕博利珠单抗联合化疗组显著改善OS（HR=0.64），且不论PD-L1 TPS的表达水平，其中TPS < 1%的人群中，死亡风险降低39%（HR=0.61）; TPS 1%-49%死亡风险降低43%（HR=0.57）; TPS≥50%的人群，死亡风险降低43%（HR=0.64），应用帕博利珠单抗联合化疗组同样改善了PFS（HR=0.56）和完全缓解率（overall respsnoe rate, ORR），且缓解更持久，两组的不良事件发生率及严重程度基本相似，研究数据建议帕博利珠单抗联合卡铂与紫杉醇或白蛋白结合型紫杉醇作为一线治疗转移性鳞癌NSCLC新的标准方案，而不必考虑PD-L1的表达水平^[15]^。基于KEYNOTE407的研究结果，2018年美国FDA批准了帕博利珠单抗联合卡铂与紫杉醇或白蛋白紫杉醇一线治疗晚期鳞状NSCLC。2019年我国CSCO指南将帕博利珠单抗联合紫杉醇和铂类（1A类证据）作为无驱动基因、鳞癌NSCLC一线治疗的Ⅱ类推荐写入指南。

IMpower150临床试验探索在抗血管生成靶向治疗结合传统化疗的基础上联合免疫治疗，是否能进一步提高疗效。研究采用PD-L1单抗阿特珠单抗联合化疗（卡铂和紫杉醇）联合或不联合贝伐珠单抗治疗用于一线治疗晚期非鳞NSCLC的疗效和安全性，患者随机分配至3个治疗组：阿特珠单抗+卡铂+紫杉醇（ACP组），或阿特珠单抗+贝伐珠单抗+卡铂+紫杉醇（ABCP组），或贝伐珠单抗+卡铂+紫杉醇（BCP组）。ABCP组合疗法，在野生型意向治疗（ITT-WT）患者群中，与BCP疗法相比，降低患者死亡风险22%，ABCP组患者的mOS为19.2个月，显著优于BCP组的14.7个月（HR=0.78）^[16]^。基于上述研究结果，FDA已批准Aezolizumab与贝伐单抗，紫杉醇和卡铂构成的ABCP组合疗法，作为一线疗法治疗转移性非鳞状NSCLC。由于ABCP组有57%的患者出现了3度-4度不良反应，这提示在实际使用该治疗方案时，需要充分评估患者可能的获益及潜在风险，谨慎作出选择，同时进一步探索与疗效及不良反应有关的分子标志物，实现个体化治疗。此外由于我国的医疗体系与国外有所不同，这一联合方案在我国是否符合卫生经济学评价原则，尚需要更多探索。

#### 4.1.2 晚期NSCLC二线免疫治疗

见表 2。

**2:** 晚期NSCLC二线免疫治疗 Second-line immunotherapy for advanced NSCLC

分层	一级推荐	二级推荐	三级推荐
既往无PD-(L)1抑制剂治疗	PD-L1未知或者无论表达状态如何：纳武利尤单抗单药	PD-L1≥1%：帕博利珠单抗单药	PD-L1未知或者无论表达状态如何：阿特珠单抗单药
既往有PD-(L)1抑制剂治疗	既往PD-(L)1抑制剂治疗：含铂两药联合化疗方案（根据组织学类型选择合适的化疗方案）		
	既往PD-(L)1抑制剂联合化疗治疗：多西他赛或其他单药化疗（一线未曾接受过的药物）		

主要证据来源：（1）纳武利尤单抗单药（CM-017、CM-057研究），不论PD-L1表达，FDA与NMPA批准; （2）帕博利珠单抗单药（KN-001、KN-010研究），PD-L1≥1%，FDA批准; （3）阿特珠单药（POPLAR、OAK），不论PD-L1表达，FDA批准。

KEYNOTE-001临床试验帕博利珠单抗治疗晚期NSCLC的疗效与安全性进行研究。数据显示至少50%的肿瘤细胞出现PD-L1表达且与帕博利珠单抗的疗效提高具有相关性，且治疗时所发生的副反应在可接受的范围内^[17]^。在此研究基础上，KEYNOTE-010研究纳入PD-L1表达阳性（TPS≥1%）且既往接受过至少一种化疗方案的局部晚期或转移性NSCLC患者，对比帕博利珠单抗与化疗药物多西他赛的临床疗效。研究结果显示，无论是帕博利珠单抗标准剂量2 mg/kg组还是高剂量10 mg/kg组的OS，均明显优于多西他赛组（10.4个月*vs* 12.7个月*vs* 8.5个月）。进一步亚组分析的结果显示，PD-L1表达水平 > 50%的患者在应用帕博利珠单抗时，其缓解率明显升高，OS明显延长。而在弱阳性组中，同样也观察到了OS的获益，但获益程度相对较小^[18]^。基于上述研究，FDA批准了帕博利珠单抗二线治疗既往接受过至少一种化疗的PD-L1≥1%的局部晚期或转移性NSCLC患者。

CheckMate-017与CheckMate-057两项Ⅲ期临床研究结果奠定了纳武利尤单抗在治疗晚期鳞癌NSCLC与晚期非鳞癌NSCLC上的疗效。纳武利尤单抗是完全人源化的抗PD-1 IgG4单克隆抗体，用于二线治疗接受过含铂化疗方案治疗的驱动基因阴性的患者，3 mg/kg，1次/2周。在针对晚期鳞癌NSCLC治疗的临床研究中，纳武利尤单抗单药改善患者的mOS（9.2个月*vs* 6.0个月，HR=0.59）^[19]^。在针对晚期非鳞癌NSCLC治疗的临床研究中，纳武利尤单抗单药相较于多西他赛改善患者的mOS（12.2个月*vs* 9.4个月，HR=0.73）^[20]^。且2项研究中≥3级的不良反应的发生率纳武利尤单抗明显低于化疗组^[21]^。目前美国FDA及我国NMPA均批准纳武利尤单抗用于治疗突变基因阴性的晚期非鳞癌NSCLC的二线治疗，且不论PD-L1的表达水平。

POPLAR研究（Ⅱ期）^[22]^和OAK研究（Ⅲ期）^[23]^分别评估了PD-L1抗体阿特珠单抗对比多西他赛，二线治疗复发性局部晚期或转移性NSCLC的患者的疗效和安全性。研究显示与传统的多西他赛治疗组相比阿特珠单抗可以显著提高患者的mOS，由9.6个月显著延长至13.8个月，死亡风险下降27%，且无论PD-L1表达水平的高低患者均能获益，但PD-L 1高表达者获益更加明显。基于上述研究，FDA批准阿特珠单抗单药二线治疗晚期NSCLC，且无论PD-L1的表达水平。

#### 4.1.3 晚期NSCLC三线免疫治疗

见表 3。

**3:** 晚期NSCLC三线免疫治疗 Third-line immunotherapy for advanced NSCLC

二级推荐
纳武利尤单抗单药治疗

主要证据来源：纳武利尤单抗单药（CA209-003研究）：CA209-003Ⅲ临床试验中，纳武利尤单抗相比于多西他赛明显改善了经治的晚期NSCLC患者的总生存。

该试验研究了经治的晚期NSCLC患者接受纳武利尤单抗治疗的Ⅰ期临床研究的随访结果，并描述了5年生存患者的特征。数据显示经评估所有接受纳武利尤单抗治疗的患者，5年生存率为16%（*N*=129）; 其中鳞癌（16%）与腺癌（15%）患者的生存率相似^[24]^。研究显示出部分经治的晚期NSCLC患者使用纳武利尤单抗能取得长期以及持续的疗效，且长期生存者具有不同的基线与治疗的特征。

#### 4.1.4 Ⅲ期不可切除的NSCLC免疫治疗

见表 4。

**4:** Ⅲ期不可切除的NSCLC免疫治疗 Immunotherapy for stage Ⅲ non-resectable NSCLC

分层	三级推荐
适合放化疗	根治性同步放化疗→度伐利尤单抗巩固治疗

主要证据来源：度伐利尤单抗用于不可切除Ⅲ期NSCLC同步放化疗后的巩固治疗（PACIFIC研究），FDA和NMPA批准。

PACIFIC研究：对接受含铂疗同步放疗后未发生疾病进展的Ⅲ期不可切除的NSCLC患者，接受ICIs剂巩固治疗的Ⅲ期临床试验。试验入组患者在完成同步放化疗且达到SD以上疗效之后，接受PD-L1抑制剂度伐利尤单抗对比安慰剂治疗，持续12个月，平均随访时间是25.2个月。结果显示度伐利尤单抗组的24个月的生存率相较于安慰机组为66.3% *vs* 55.6%，显著延长了患者的总生存时间^[25]^。PACIFIC临床试验数据确定了度伐利尤单抗在局部晚期（Ⅲ期）NSCLC的治疗地位，美国FDA批准度伐利尤单抗用于不可切除Ⅲ期NSCLC同步放化疗后的巩固治疗。

### 4.2 驱动基因敏感突变阳性NSCLC

对于EFGR/ALK阳性的NSCLC进行免疫治疗目前尚缺乏充分证据，在Impower 150研究的亚组分析结果显示以下方案具有一定效果：阿特珠单抗+贝伐珠单抗+卡铂+紫杉醇。

主要证据来源：IMpower 150研究亚组分析中EFGR/ALK阳性人群可以从阿特珠单抗联合化疗（卡铂和紫杉醇）联合贝伐珠单抗更大获益。

在IMpower150研究中，纳入的ITT人群中包括108例*EGFR*突变或*ALK*易位患者。对这群患者进行的亚组生存分析显示，阿特珠单抗+贝伐+化疗组的mPFS较贝伐联合化疗组更长（9.7个月*vs* 6.1个月，HR=0.59）^[16]^。IMpower150是第一个采用ICIs在*EGFR/ALK*突变患者中显示出有临床获益的随机Ⅲ期研究。在贝伐+化疗的标准基础上增加ICIs，对于*EGFR/ALK*突变的晚期NSCLC患者，该方案有可能成为一种新的治疗选择。

#### 4.2.1 生物标志物

PD-L1表达：目前认为肿瘤组织PD-L1的表达是抗PD-1/PD-L1治疗前选择优势人群比较合理的标志物。KEYNOTE-024研究结果显示帕博利珠单抗在PD-L1表达≥50%的驱动基因阴性的晚期NSCLC人群中，一线治疗效果优于化疗^[11]^。KEYNOTE-042研究进显示帕博利珠单抗能显著改善PD-L1表达≥1% NSCLC患者的mOS^[12]^。2项临床研究均证实了PD-L1表达水平与免疫治疗疗效的相关性。CheckMate-057研究对比了纳武利尤单抗单药与多西他赛二线治疗NSCLC的疗效，无论PD-L1的表达水平，免疫治疗相较于化疗均能获益，但在PD-L1低表达或者不可检测的患者中，研究人员未观察到相似的OS获益^[20]^。因此PD-L1是晚期NSCLC的免疫治疗疗效预测的生物标志物之一。

#### 4.2.2 肿瘤基因突变负荷（tumor mutational burden, TMB）/血液肿瘤基因突变负荷（blood tumor mutational burden, bTMB）

目前研究显示TMB/bTMB作为ICIs治疗效果的预测标志物尚存在较大争议。在CheckMate-026^[26]^和POPLAR^[22]^/OAK^[23]^研究的探索性分析中提示高TMB/bTMB患者能从免疫治疗中获益。但在KEYNOTE系列研究的探索性分析的结果显示tTMB与疗效无相关性，无论tTMB的高或低，帕博利珠单抗+化疗在鳞状和非鳞状NSCLC患者的一线治疗中均显示出生存获益^[27]^。

#### 4.2.3 错配修复缺陷（mismatch repair deficient, dMMR）或微卫星不稳定-高（microsatellite instability-high, MSI-H）

有研究报道基于错配修复（mismatch repair, MMR）表达水平指导临床应用帕博利珠单抗治疗晚期肿瘤，预测帕博利珠单抗的临床疗效^[28]^。CheckMate-142临床研究评估纳武利尤单抗单药与纳武利尤单抗联合伊匹木单抗治疗转移性结直肠癌的效果，在MSI-H的结直肠癌患者中，单药治疗组和联合治疗组患者的ORR优于微卫星稳定患者^[29]^。MMR状态虽然有可能用于预测PD-1/PD-L1抑制剂的疗效，但由于其在肺癌中的发生率很低，dMMR/MSI-H对肺癌免疫治疗疗效的预测价值还需要更多的研究和数据来验证。

### 4.3 晚期NSCLC的治疗路径图

见图 1。

**1:**
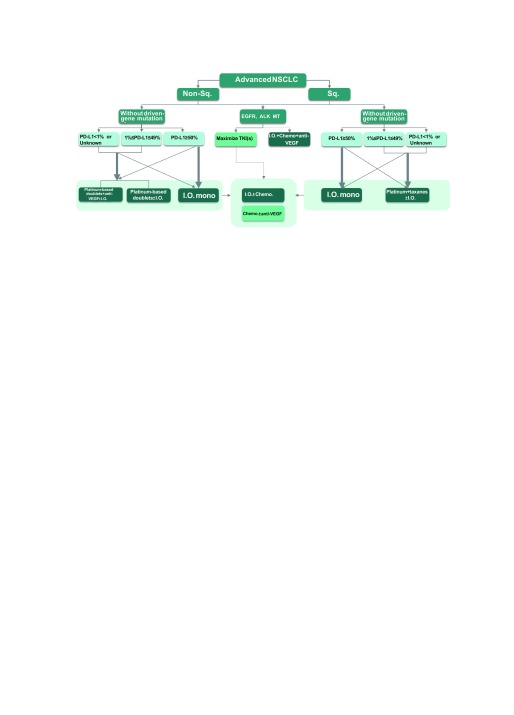
晚期NSCLC的治疗路径 Treatment pathways for advanced NSCLC. Non-Sq.: non-squamous; Sq.: squamous; EGFR: epidermal growth factor receptor; ALK: anaplastic lymphoma kinase; MT: mutation; TKI: tyrosine kinase inhibitors; Chemo: chemotherapy; I.O. mono: immuno monotherapy

## 5 免疫治疗的评估

目前，对于肿瘤治疗的疗效评价，通常采用实体肿瘤疗效评估标准1.1（Response Evaluation Criteria In Solid Tumors, RECIST1.1），其主要依据影像学上肿块大小的变化作为判定标准，但是可能会低估免疫治疗对患者的获益。

2017年初，RECIST工作组正式提出实体肿瘤免疫疗效评价标准（Modified RECIST 1.1 for immune based therapeutics, iRECIST）^[30]^，iRECIST标准引入了即待证实的疾病进展（immune unconfirmed progressive disease, iUPD）和已证实的疾病进展（immune confirmed progressive disease, iCPD）的概念，而将之前RECIST 1.1标准评定的PD暂视为iUPD，依据患者的肿瘤类型、疾病分期和临床情况综合判断是否继续治疗，在4周-6周进行再次评估以确认iCPD。在此评价模式下，iUPD之后可分别出现病情稳定（immune stable disease, iSD）、部分缓解（immune partial response, iPR）或免疫完全缓解（immune complete response, iCR）等几种可能，只要iCPD未得到证实，就要循环持续评价并记录未证实的原因。iRECIST标准提出了循环反复评价的模式，一定程度上可捕获免疫治疗时代下非典型缓解类型（如假性进展和延迟反应）的出现。

为了探究iRECIST疗效评价标准在接受PD-1/PD-L1抑制剂的NSCLC患者中接受疗效评价的差别，已有研究团队开展了一项回顾性分析，应用新老评价标准分别对患者的短期疗效结果进行评价。从2013年2月-2016年12月，共有160例患者纳入该回顾性分析。最终，共有20例患者（13%）确认为不典型应答，其中8例患者（5%）为假性进展（肿瘤先增大后缩小），12例（8%）患者呈现分离性应答（混合型反应，即某些肿瘤增大，另一些却缩小）。这些患者的OS明显优于确定进展的患者。有13例（11%）患者根据RECIST 1.1评价标准评估为PD，但却最终确认为临床获益。根据RECIST 1.1进行评价，37例（23%）为应答或SD，123例（77%）为PD。但是根据iRECIST疗效评价标准，80例可评价，15例患者难以进行评估。NSCLC患者接受免疫治疗时，可能会发生13%左右的不典型应答，其中包括假性进展和分离性应答，这些患者的OS明显优于真正确认为PD的患者。约11%接受免疫治疗超过6个月患者可能被RECIST 1.1标准评价为PD，其实却能够从治疗中获益^[31]^。

## 6 免疫治疗的不良反应

以ICIs为代表的免疫治疗改变了肿瘤治疗的格局，但免疫治疗在延长患者生存期的同时也带来了药物不良反应等问题，尤其是免疫治疗所特有的不良反应即免疫相关不良反应（immune-related adverse events, irAEs）^[32, 33]^。虽然irAEs的总体发生率较低，但有些irAEs可导致严重后果^[34]^，需要高度重视和积极防治。

### 6.1 irAEs的发生机制

irAEs的发生可能与ICIs改变了机体的免疫状态有关。CTLA-4通路在T细胞反应的早期阶段（免疫致敏阶段）起抑制作用，激活中央淋巴组织的T细胞，同时影响Treg细胞的功能。因此CTLA-4抗体引起的irAEs比较广泛、发生率较高，特异性较小，毒性较强。抗PD-1/PD-L1抗体在T细胞反应后期（免疫效应阶段）发挥作用，主要多激活外周组织中的T细胞（如肿瘤微环境），因此引起的irAEs比较局限，发生率较低，特异性较强，毒性相对弱。由于肿瘤免疫治疗可以增加机体自身的免疫系统活性，所以ICIs除作用于肿瘤细胞之外，也会潜在地对健康组织产生毒性作用，从而引起其他系统的irAEs^[35]^。

### 6.2 irAEs的处理原则

2018年美国临床肿瘤学会（American Society of Clinical Oncology, ASCO）联合NCCN共同发布了免疫治疗相关不良反应管理指南以指导临床实践^[36]^，2019年CSCO发布了免疫检查点抑制剂毒性管理指南^[37]^。

irAEs的基本处理原则包括：预防、检测、评估、治疗和监控。

#### 6.2.1 预防

对患者及其家属做好治疗开始前、治疗过程中以及治疗后生存期内的与治疗相关的不良反应的教育。注意是否存在自身免疫性疾病的既往史和家族史。临床医师必须熟悉irAEs的特点和危险因素，密切关注使用ICIs后是否出现不适或原有一些症状的加重，早期识别和处理可减少irAEs的持续时间和严重程度^[38]^。

#### 6.2.2 检测

在患者治疗开始前进行病史询问、体格检查、实验室检查及影像学检查作为基线参考对判断是否可能出现irAEs尤为重要。当患者用药后出现新的症状，或原有症状加重，应完善体格检查、实验室检查、影像学检查等，必要时进行其他相关检查后进行评估。

#### 6.2.3 评估

当患者用药后出现新症状或原有症状加重，可能为疾病进展、偶然事件或出现irAEs，而且癌症患者在使用ICIs前可能已合并一些基础疾病，因此关注已有的症状，并根据患者基线时的特殊病史、症状或伴随疾病等，与基线值对比，判断是否为irAEs并评估其严重程度。

#### 6.2.4 治疗

irAEs的总体处理原则和irAEs的级别有关，按处理原则来分，总体分为以下几种情况。

##### 6.2.4.1 一级irAE毒性反应

出现一级毒性反应时，一般均可在密切监测下继续治疗，但是神经系统及一些血液系统的毒性反应除外。

##### 6.2.4.2 二级irAE毒性反应

出现大部分二级毒性反应时，应当停止治疗，直到症状和/或实验室指标恢复到一级毒性反应或更低水平; 可给予糖皮质激素（初始剂量为泼尼松0.5 mg/kg/d-1 mg/kg/d或等剂量的其他激素）。

##### 6.2.4.3 三级irAE毒性反应

出现三级毒性反应，应当停止治疗，并且立即使用高剂量糖皮质激素（泼尼松1 mg/kg/d-2 mg/kg/d，或甲泼尼龙1 mg/kg/d-2 mg/kg/d）。糖皮质激素减量应持续4周-6周以上; 对于某些毒性反应如果使用高剂量糖皮质激素48 h-72 h后症状没有改善，可选择英夫利昔单抗（Infliximab）。当症状和/或实验室指标恢复到一级毒性反应或更低水平，可以恢复治疗，但应慎重，尤其是对于治疗早期就出现不良事件的患者，同时不推荐进行剂量调整。

##### 6.2.4.4 四级irAE毒性反应

出现四级毒性反应，一般意味着永久停止治疗，已用激素替代疗法控制的内分泌不良事件除外。

irAEs发生的时间和累及器官有关，一般在给药后几周至几月内发生，但实际上是irAE可发生于接受ICIs治疗的任何时间，甚至是延迟到ICIs治疗结束后。

##### 6.2.4.5 NSCLC常见irAEs

一项针对不同肿瘤组织类型及采用不同ICIs治疗后irAEs表现与发生率差异的系统性分析研究显示。在ICIs治疗中，最常见的irAEs多累及内分泌器官（如甲状腺功能减退、甲状腺功能亢进、垂体和肾上腺功能障碍）、胃肠道（如腹泻、结肠炎和恶心）、肺脏（如肺炎）、皮肤（如皮疹、瘙痒和白癜风）和骨骼肌肉系统（如关节痛和肌痛）。对ICIs治疗黑色素瘤与NSCLC irAEs的比较分析显示，结肠炎、腹泻、瘙痒、皮疹等胃肠道与皮肤irAEs的发生率在黑色素瘤中较高，而肺炎等肺部irAEs的发生率在NSCLC中较高，这可能与NSCLC患者合并慢性阻塞性气道疾病或接受过肺部放疗等既往治疗有关^[39]^。

### 6.3 irAEs的分级和主要处理原则^[40, 41]^

见表 5。

**5:** irAEs的分级和主要处理原则^[40, 41]^ irAEs classifications and main principles of management^[40, 41]^

irAEs的分级及主要处理原则			
irAEs	分级标准	治疗原则	随访后治疗
腹泻/肠炎	1级：腹泻 < 每天4次	①继续ICIs治疗 ②对症（如止泻等）	密切监测症状
	2级：腹泻≥每天4次-6次; 出现腹痛、便血等	①延迟ICIs治疗 ②对症	①如果改善至1级; 恢复ICIs治疗 ②如果5 d后不缓解或加重：0.5 mg/kg/d-1.0 mg/kg/d泼尼松龙口服或等效静脉给药： a.若改善至1级则类固醇减量，至少服用1个月 b.若无改善则按照3/4级处理，可考虑胃肠镜检查及活检
	3级：腹泻≥每天7次; 剧烈腹痛，腹膜征等	①停用ICIs ②补液、营养等对症治疗 ③ 1.0 mg/kg/d-2.0 mg/kg/d泼尼松龙口服或等效静脉给药; 预防性使用抗生素防止机会性感染 ④考虑胃肠镜检查	①若改善:继续激素治疗直至恢复到1级，然后激素逐渐减量至少1个月; ②如果2 d-3 d后无改善:加用英夫利昔单抗5 mg/kg (除外败血症或穿孔等禁忌症)
	4级：胃肠穿孔等	①紧急按3级处理 ②内外科共同治疗	永久停用ICIs
肝炎	1级：AST/ALT > 3倍正常值上限(ULN)	①继续ICIs治疗 ②查HAV、HBV、HCV、CMV等指标，除外肝脏基础疾病 ③限酒	①监测肝功能 ②如果加重：按照2级或3/4级的方法治疗
	2级：AST/ALT: 3倍-5倍ULN	①延迟ICIs治疗 ② 0.5 mg/kg/d-1.0 mg/kg/d泼尼松龙口服或等效剂量静脉	①每3天监测肝功能; 如果好转激素减量，至少1个月，逐渐恢复治疗 ②如果无改善或加重，继续激素治疗
	3级：AST/ALT: 5倍-20倍ULN	①终止ICIs治疗 ② 1.0 mg/kg/d-2.0 mg/kg/d泼尼松龙口服或等效剂量静脉给药	①如果恢复到2级:类固醇减量，至少1个月 ②如果未改善，加重或反弹:加用麦考酚酯等免疫抑制剂
	4级：AST/ALT > 20倍ULN	处理同3级	永久停ICIs治疗
皮疹	1级：皮疹 < 10%体表面积	①对症治疗（如抗组胺药、外用类固醇激素） ②继续ICIs治疗	观察
	2级：皮疹占10%-30%体表面积	①可忍受：同1级 ②难以忍受:口服0. 5 mg/kg/d-1. 0 mg/kg/d泼尼松龙1-2周 ③延迟ICIs治疗 ④如果持续＞1周-2周或复发：考虑皮肤活检	①若改善，类固醇减量至少1个月 ②若加重按照3级-4级的方法治疗
	3级：皮疹 > 30%体表面积	①延迟或终止ICIs治疗 ②考虑皮肤活检 ③皮肤科会诊 ④ 1.0 mg/kg/d泼尼松龙口服或等效剂量静脉给药	①如果改善至1级：类固醇减量至少1个月
	4级：当前没有定义	同3级	永久停用ICIs治疗
肺炎	1级：仅在X线上有改变	①考虑延迟ICIs治疗 ② 1.0 mg/kg/d泼尼松龙口服或等效剂量静脉	3周后重新评估: a.如果缓解继续ICIs治疗; b.如果加重按2，3-4级处理
	2级：轻度至中度新发症状	①延迟ICIs治疗 ②对症 ③ 1.0 mg/kg/d-2.0 mg/kg/d泼尼松龙口服或等剂量静脉给药，经验抗生素治疗	每1-3天重新进行评估: a.如果改善，激素减量至少1个月并继续ICIs治疗 b.如果2周后未改善或加重，按照3级或4级的方法治疗
	3级：严重的新发症状; 新发或加重缺氧	①终止ICIs治疗 ②对症 ③ 2 mg/kg/d-4 mg/kg/d甲强龙口服或等效剂量静脉给药; 预防性抗生素抗感染 ④考虑支气管镜检查、肺活检	每天进行评估: a.如果改善至基线水平：逐步减少类固醇剂量至少6周; b.如果48 h后未改善或加重：加用其他免疫抑制(如英夫利昔单抗、麦考酚酯、静脉免疫球蛋白等)
	4级：威胁生命	①抢救如气管插管 ②重症监护	永久停止ICIs治疗
甲亢/甲减	无症状TSH升高	继续ICIs治疗	监测甲功
	症状性内分泌病变	①延迟ICIs治疗 ② 1 mg/kg/d-2 mg/kg/d泼尼松龙口服等效剂量静注给药 ③激素替代治疗:甲减使用左旋甲状腺素; 甲亢使用心得安及卡比马唑 ④内分泌科会诊	监测甲功: a.若改善:类固醇减量至少1个月，依照研究方案恢复ICIs治疗
肾炎	1级：肌酐 > 1-1.5倍基线水平; 尿蛋白：+，定量 < 1.0 g/d	①继续ICIs治疗 ②监测肾功能 ③水化 ④停肾损药物	a.如果恢复:继续原治疗 b.如果加重:按照2级或3/4级的方法治疗
	2级：肌酐1.5-3倍基线水平尿蛋白：++，定量1.0 g/d-3.4 g/d	①延迟ICIs治疗 ②除外非免疫性肾损害 ③ 0.5 mg/kg/d-1.0 mg/kg/d泼尼松龙	a.如果返回至1级:类固醇减量至少1个月，恢复ICB治疗、监测肾功能 b.如果不缓解或加重:永久停用ICB，3级-4级必要时可行透析治疗
	3级：肌酐 > 3倍基线水平尿蛋白：定量≥3.5 g/d	①停止ICIs治疗 ② 1.0 mg/kg/d-2.0 mg/kg/d泼尼松龙口服或等效剂量静脉 ③考虑肾穿刺活检	
	4级：肌酐 > 6倍基线水平	同3级	
ALT: Alanine aminotransferase; AST: Aspartate aminotransferase; TSH: thyroid stimulating hormone.			

#### 6.3.1 严密监测

因为irAEs可发生于治疗全程以及治疗结束后，因此随访监测很重要。推荐治疗结束后第一年每月，之后每6个月进行随访评估，与基线数值进行对比，若怀疑发生irAEs，及时按上诉治疗原则进行处理。同时，注意激素不良反应的管理，如预防机会性感染、补钙以及护胃等处理。

#### 6.3.2 irAEs恢复后再次接受免疫治疗的原则

影响患者再次使用ICIs的关键因素取决于末次irAEs出现的程度、患者的一般状况以及是否存在其它治疗模式。如果二级irAEs的症状和/或实验室指标降至一级或以下，可以考虑再次使用ICIs，但是需要谨慎使用，特别对于出现早发irAEs的患者，不推荐剂量调整。严重irAEs缓解后，患者再次接受ICIs治疗，irAEs可能再次出现; 如果再次出现irAEs，则这类ICIs应永久停用。

#### 6.3.3 免疫治疗超进展（hyperprogression, HP）

HP是相对于通常的进展而言，HP被定义为肿瘤反常的加速生长，包括：①在ICIs治疗后第一次评价时出现进展，或至治疗失败时间（TTF）2个月; ②肿瘤体积增加 > 50%;③肿瘤增长速度（TGR）增加 > 2倍。目前关于HP的机制尚不明确。HP可发生于ICIs治疗过程中任何阶段，且无明确的预测指标，初步估计达10%，对于老年患者更需关注。尚未发现HP与肿瘤负荷、肿瘤类型、治疗线数、PD-L1表达水平之间存在相关性。发生HP的患者总体预后较差，OS仅为3个月-4个月; 一旦出现HP的症状，需尽早由免疫治疗转为抢救化疗^[42, 43]^。

## 7 免疫治疗的相对禁忌证

### 7.1 自身免疫性疾病

一直以来，合并自身免疫性疾病的患者，都是ICIs使用的相对禁忌人群。不过，患有自身免疫性疾病的患者，可根据疾病控制的现状分为三类：①病情处于活动期; ②目前处于治疗期且病情得到控制; ③目前未治疗但病情已得到控制。有研究对上述三种情况的患者进行了研究，汇总了56例NSCLC合并自身免疫性疾病的患者接受PD-1抗体治疗的最新研究，总体来说，接受PD-1抗体治疗后，发生自身免疫病的反弹或爆发的绝大多数都是第一类患者，且大部分患者需要激素治疗才能控制^[44]^。因此，对于合并自身免疫病的患者，如果病情已经控制得当，接受PD-1抗体的治疗，总体而言是安全的。但是，对于病情尚未控制的人群，使用PD-1抗体大概率会加重病情。

### 7.2 体弱和高龄

患者一般从临床经验而言，患者的体力活动状态评分（performance status, PS）0分-1分的患者可以耐受放化疗; 2分的患者可考虑接受靶向药物治疗或者ICIs治疗; 3分-4分的患者则需要慎重接受抗癌治疗。

近期，有学者分析报告了高龄（> 70岁）、体弱（PS 2分）的肿瘤患者，接受PD-1抗体治疗的情况。数据显示在安全性方面无差异：3级-5级严重不良反应发生率，总人群为6%，高龄患者为6%，体弱患者为9%，无统计学差异。治疗相关不良反应的发生率，总人群为37%，高龄患者为38%，体弱患者为29%，无统计学差异。在生存获益方面，总人群mOS为9.1个月，高龄患者mOS为10.4个月，无统计学差异。两组的2年生存率分别为26%和25%，也没有差异。但是，对体弱患者而言，相比于PS 0分-1分的患者，其mOS只有4.0个月，2年生存率仅为9%^[45]^。综上所述，年龄 > 70岁，并不是使用PD-1抗体的禁忌证。单纯的高龄可能并不显著影响PD-1抗体的疗效和安全性。不过，体力与体能状态较差的患者，接受PD-1抗体治疗后生存期可能会缩短。

### 7.3 长期使用激素者

已有研究表明基线时使用皮质类固醇（≥10 mg泼尼松）与免疫治疗的疗效相关。有研究分析了640例接受单药PD-L1抑制剂治疗的晚期NSCLC患者，其中90例患者（占14%）在接受PD-L1治疗前就已开始使用皮质类固醇（≥10 mg泼尼松），分析显示这14%接受皮质类固醇（≥10 mg泼尼松）治疗的患者的OS和PFS明显变差^[46]^。目前，尚不清楚这些患者疗效变差是否与使用皮质类固醇的免疫抑制作用直接相关，但我们建议在开始免疫治疗时谨慎使用皮质类固醇，除非必须进行激素治疗（如脑转移）。

### 7.4 HIV感染患者

HIV病毒感染会摧毁机体的免疫系统，因此多种肿瘤的发生率明显提高。另一方面，PD-1抗体本身并不能直接杀死癌细胞，而是通过激活人体的免疫系统发挥作用，因此PD-1抗体需要一个基本完整的免疫系统，才能发挥抗肿瘤的疗效，因此一般认为，感染HIV病毒是免疫治疗的禁忌证。有研究对30例合并HIV病毒感染的癌症患者接受PD-1抗体治疗的相关情况进行了研究，结果显示，在安全性方面，经过PD-1抗体治疗后，22例患者出现了1级-2级较轻微不良反应，6例患者出现了3级不良反应，而不良反应的表现形式与普通人群基本相同，包括乏力、甲减、恶心、皮疹、肺炎等。在疗效方面：1例肺癌患者肿瘤完全消失、2例淋巴瘤患者肿瘤明显缓解、2例卡西波肉瘤患者疾病稳定，总体的抗癌疗效和普通人群无太大差异。治疗期间HIV病毒数量没有出现明显的反弹，病情未处于活动，但是CD4^+^ T细胞数目也没有出现明显的恢复^[47]^。所以，对于病情基本控制的HIV感染患者来说，使用PD-1抗体治疗的疗效和毒副作用与非感染患者基本相似。
