# 更正声明

**DOI:** 10.3779/j.issn.1009-3419.2020.101.14

**Published:** 2020-03-20

本刊2020年第23卷第2期刊登的题为“中国非小细胞肺癌免疫检查点抑制剂治疗专家共识（2019年版）”[周彩存, 王洁, 步宏, 等. 中国非小细胞肺癌免疫检查点抑制剂治疗专家共识(2019年版). 中国肺癌杂志, 2020, 23(2): 65-76.] 一文中：第65页作者单位中“400042 重庆，重庆大坪医院（朱波）”应改为“400037 重庆，重庆新桥医院（朱波）”；第66页第1部分第3行“年龄标化率（age standardized rate, ASR）1.5/10万和14.6/10万”应改为“年龄标化率（age standardized rate, ASR）31.5/10万和14.6/10万”。特此更正。对此深表歉意。

Erratum: Chinese Experts Consensus on Immune Checkpoint Inhibitors for Non-small Cell Lung Cancer (2019 version) 

Zhou CC, Wang J, Bu H, *et al*. 

Zhongguo Fei Ai Za Zhi, 2020, 23(2): 65-76.

In the version of this article initially published, error appeared on page 65. The author address should be “400037 重庆，重庆新桥医院（朱波）”. And another error appeared on page 66. The first sentence in part 1 should be “年龄标化率（age standardized rate, ASR）31.5/10万和14.6/10万”.

## Notes

https://www.ncbi.nlm.nih.gov/pmc/articles/PMC7049793

